# Utilization of Vestibular Information for Balance Control in Children with Chiari I Malformation

**DOI:** 10.3390/audiolres14060079

**Published:** 2024-10-31

**Authors:** Irene Stella, Philippe Perrin, Matthieu Casteran, Anthony Joud, Art Mallinson, Olivier Klein

**Affiliations:** 1 Department of Paediatric Neurosurgery, University Hospital of Nancy, 54011 Vandoeuvre-lès-Nancy, France; 2 Research Unit DevAH—Development, Adaptation and Handicap, Faculty of Medicine, University of Lorraine, 54511 Vandoeuvre-lès-Nancy, France; philippe.perrin@univ-lorraine.fr (P.P.); matthieu.casteran@univ-lorraine.fr (M.C.); 3 Laboratory for the Analysis of Posture, Equilibrium and Motor Function (LAPEM), University Hospital of Nancy, 54011 Vandoeuvre-lès-Nancy, France; 4 Department of Paediatric Otolaryngology, Children Hospital, University Hospital of Nancy, 54500 Vandoeuvre-lès-Nancy, France; 5 Faculty of Science and Techniques of Physical and Sport Activities, University of Lorraine, 54600 Villers-lès-Nancy, France; 6 Research Unit 2LPN, Psychology and Neuroscience Lab, University of Lorraine, 54000 Nancy, France; 7 Division of Otolaryngology, Department of Surgery, Faculty of Medicine, University of British Columbia, Vancouver, BC V6T 1Z4, Canada; art@mallinson.ca

**Keywords:** Chiari I malformation, pediatric neurosurgery, posturography, balance

## Abstract

(1) Background: Surgery for Chiari I malformation (CMI) is indicated when typical clinic-radiological features (syringomyelia, exertional headaches, sleep apnea syndrome, and tetraparesis) are present. Sometimes, patients have atypical complaints suggestive of otolaryngological (ENT) involvement, and it is sometimes difficult for the neurosurgeon to determine if these complaints are related to the CMI. Our aim was to describe postural control patterns in children with CMI using computerized dynamic posturography. To our knowledge, this is the first study addressing postural instability in pediatric CMI patients. (2) Methods: Twenty-eight children aged 6 to 17 years with both radiologically confirmed CMI and clinical ENT complaints were included. The children were separated into two groups, operated and non-operated patients, based on neurosurgical indication. Epidemiologic and posturographic results (CDP—Equitest^®^) were compared between both groups, as well as pre- and postoperatively in Group 2. (3) Results: In Group 2 patients, significant improvement of global SOT was found after intervention. When the three sensorial aspects of postural control calculated by the system were independently assessed, the greatest improvement was in the vestibular ratio. We also observed an altered CoG pattern (“lateral deviation”) in the Group 2 patients, which significantly differed from those in Group 1. Lateral deviation was significantly reduced postoperatively in the Group 2 patients. A correspondence between preoperative MRI and the side of lateralization on posturography was found in four children, but this cannot be regarded as significant due to the low number of patients. (4) Conclusions: Postural control seems to improve after surgery for CMI in children, mostly due to the improvement in vestibular function. There is a correspondence between the side of lateral deviation and the side of greatest tonsillar descent on MRI and perioperatively. Further studies are needed to support these results and to confirm the utility of CDP in CMI patients.

## 1. Introduction

Initial reports about audiovestibular symptoms in patients with Chiari I malformation (CMI) date back to 1971, when Rydell and Pulec [1] described a series of 29 patients presenting otoneurological symptoms in a population of 130 subjects with CMI, explored at the Mayo Clinic (Rochester, MN, USA). The most frequent symptoms were hearing loss, tinnitus, vertigo, and imbalance. Imbalance was reported as the chief clinical complaint in 30% of adult CMI patients in another paper published soon after by the same group [2]. Since that time, otolaryngologists have maintained their interest in exploring audiovestibular involvement in CMI, stressing the importance of this relationship.

Chiari I malformation (CM1) is a structural abnormality characterized by cerebellar tonsillar descent of at least 5 mm below the level of the foramen magnum [3], or of Mc Rae’s line, into the vertebral canal. Once classified as a rare disease, it is increasingly diagnosed due to the improvements in cerebral imaging. Although the true prevalence in the general population is difficult to establish, the imaging incidence in children younger than 18 years has been reported to be between 0.4 to 3.6% [4,5].

The symptoms related to CMI have been classically divided into four categories [2,3,6,7], as follows:

(1) Exertional nuchal pain and headaches;
(2) Symptoms related to brainstem and lower cranial nerve dysfunction;
(3) Symptoms related to syringomyelia, occurring in 45–75% of CMI patients [2,8];
(4) Cerebellar symptoms. A clinical difference between adults and children is the increased occurrence in children of central sleep apnea syndrome (CSAS). Feeding problems and oropharyngeal symptoms are also more frequent in children under 3 years of age [4]. Traditionally, the decision about surgical management of CMI being necessary was made regarding the presence of syrinx, central apnea syndrome, or symptoms believed to be characteristic for CMI, such as exertion headache, exertional neck pain, pyramidal signs, and tetraparesis.

Although, from an anatomical point of view, it is possible that audiovestibular disorders might arise from a crowded posterior cranial fossa, where the brainstem, the flocculonodular lobe of the cerebellum, and cranial nerves may be compressed or distorted, rarely are these symptoms alone sufficient to justify surgical intervention. It is the presence of nystagmus, particularly downbeat nystagmus (DBN), that has largely been associated with CMI [1,9,10,11,12,13,14,15,16,17,18,19,20,21,22]. Although many neurosurgeons accept DBN as a presenting symptom of CMI [6,7], ENT evaluation is not systematically required prior to occipito-cervical decompression, and surgical intervention does not rely on it. In the literature, there is no consensus on the precise anatomical substrate of audiovestibular signs and symptoms seen in CMI patients, as these symptoms are classified as cerebellar in some studies [6,15], or as originating from brainstem compression or lower cranial nerves in others [3,7]. The recognition of the relationship between audiovestibular symptoms or signs and CMI become fundamental in the process of the selection of surgical candidates in patients who do not have other criteria for intervention (i.e., syringomyelia or CSAS) but have atypical headaches or, in some cases, are defined as “asymptomatic”.

Given the fact that nystagmus has already been largely described in the literature, we decided to focus our attention on imbalance, which is another frequently reported, but less studied, symptom. The term “imbalance”, also known as disequilibrium or gait ataxia, suggests a lack of movement coordination in the movements of the voluntary muscles. In the literature, it is often included as part of the general and more widely used term “dizziness”, referring to a sense of disorientation in space. “Vertigo” is a more specific term, which is defined as a sensation of rotation of oneself or of surrounding objects [23,24,25]. There are different types of ataxia that are described [26], depending on the site of lesion.

Balance disorders in CMI patients manifest with gait ataxia. To assess the real etiology of gait instability in CMI patients, it is necessary to perform a more precise investigation, as the clinical picture is often poor, and might not provide answers. One of the most quantitative and reliable methods of assessing postural control is dynamic posturography [27]. At our institution, computerized dynamic posturography (Equitest^®^, NeuroCom, Clackamas, OR, USA) (CDP) at the Laboratory for the Analysis of Posture, Equilibrium, and Motor Function (LAPEM)—University Hospital of Nancy, can be performed after ENT examination.

The aims of this study were as follows:

▪ To define the presence of imbalance in children with a diagnosis of CMI;
▪ To explore the etiologic mechanisms underlying imbalance in these patients;
▪ To determine if surgery has any impact on balance control;
▪ To explore the usefulness of ENT examination and the use of CDP in the surgical management of CMI children.

The details of our study’s protocol have been already published [28] and will be summarized in this manuscript.

To the best of our knowledge, there are few studies in the literature describing balance control patterns in CMI patients [29,30], and this is the first study about balance control analysis in a pediatric CMI cohort using Equitest^®^ (computerized dynamic posturography, or CDP).

## 2. Patients and Methods

### 2.1. Patients

Between September 2019 and September 2023, 48 children with a diagnosis of CMI were seen at our institution. The radiologic criterion for the diagnosis of CMI was the presence of a caudal displacement of cerebellar tonsils of at least 5 mm under McRae’s line [3]. The inclusion criteria for this study were the following: children aged from 6 to 18 years with radiologically confirmed CMI, presenting clinical features suggesting ENT involvement (dizziness, nystagmus, gait impairment, motion sickness, malaise, and atypical migraine, which could not be directly attributed to the CMI).

Patients with tonsillar ectopia secondary to other complex pathology (e.g., craniostenosis, craniocervical malformation, intracranial hypertension, and posterior fossa tumor) were not included in this study, as tonsillar ectopia in these cases might not fall into the diagnosis of CMI. Children with pre-existing vestibular pathology, children unable to stand on the platform (due to cerebral palsy, severe behavioral troubles, severe visual impairment, or associated orthopedic pathologies), and children and/or parents who refused to participate in this study were also excluded. In our population of 48 patients,

8 patients were not included in the study because they did not meet the inclusion criteria, as follows:

▪ A total of 2 patients were less than 6 years of age;
▪ A total of 1 patient had severe visual impairment;
▪ A total of 2 patients had cognitive impairment;
▪ A total of 2 patients were operated for craniocervical decompression for cerebellar ptosis secondary to craniostenosis;
▪ A total of 1 patient had already been operated on in the past for Chiari I malformation.

In addition, 5 patients refused to be enrolled.

**Figure 1:**
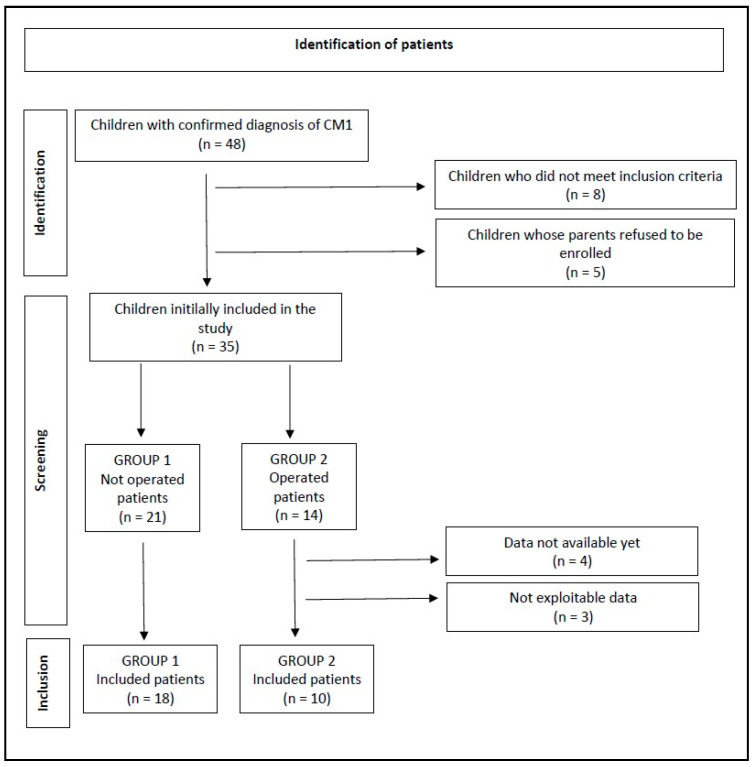
Flow chart diagram according to PRISMA 2020 guidelines. CM1: type 1 Chiari malformation.

The 35 remaining patients, who all agreed to be included in this study, were divided into two groups, independent of the ongoing results of this study. The first group included 21 patients for whom there was no indication for surgery, and the second one was a group of 14 patients who would have benefited from surgical intervention. The criteria that led the neurosurgeon to decide on surgical intervention are represented by the presence of at least one of the following aspects: characteristic symptomatology (most of all, exertional headaches, usually occipito-cervical, but also of frontal location; and presence of symptoms of brainstem compression); syringomyelia; and central sleep apnea syndrome. One operated girl missed her definitive postoperative posturographic assessment; in two cases, the results could not be analyzed; and, at the time of this paper, data are not available for four children yet. In total, data are available for 28 patients, including 18 non-operated and 10 operated ones (Figure 1).

### 2.2. Methods

All patients benefited from a complete clinical evaluation and in-depth history taking, recorded in a survey, a medullary MRI to check for syringomyelia, and a polysomnographic recording to look for sleep apnea syndrome.

The patients enrolled in this study were all referred for ENT assessment, including neuro-otological examination and CDP with Equitest^®^. This evaluation was conducted in a blind fashion, as the ENT specialist (Ph.P.) did not know whether or not the patients were surgical candidates.

The ENT assessment consisted of a neuro-otological examination and clinical vestibular assessment, if deemed necessary. The aims of the neuro-otological examination were to detect and differentiate cerebellar from vestibular signs, identify segmental or axial deviations, and rule out confounding associated factors. Other evaluated factors included vergence insufficiency, refraction disorders, and other visual corrections. Vertigo or dizziness were accurately assessed, as well as complaints of tinnitus. Otoscopic examination was also carried out, with tympanometry and acoustic reflex test recordings (Interacoustics, Middelfart, Denmark) and determining hearing thresholds (pure-tone air and bone-conduction thresholds) in tone audiometry (from 250 Hz to 8000 Hz) and intelligibility in speech tests (Interacoustics).

After that, CDP (computerized dynamic posturography) was carried out to determine balance control performances.

CDP assesses the global balance performance and relative weight of sensory information (visual, vestibular, and somatosensory) involved in balance control. The Equitest^®^ balance system consisted of a dual platform with two footplates connected with a pin joint. The footplates were supported with five force transducers. The computer calculated the center of foot pressure (CoP) and the vertical component of the center of gravity (CoG), using the subject’s height entered by the operator. When a subject stands with their ankles centered over the stripe on the dual platform, with feet an equal distance laterally from the center line, this position is called the “electrical zero position” and serves as a reference point for the calculation of sway angles. The sensory organization test (SOT) consisted of three 20 s trials under six different sensory conditions, in which the surface and/or visual surroundings (i.e., sensory inputs) were systematically manipulated (so-called “sway referencing”) [27,31,32,33]. (Table 1).

To protect against falls, the patients wore a safety harness connected to the ceiling and an operator stood within reaching distance. The equilibrium score (ES) was calculated by comparing the subject’s anterior–posterior sway during each 20 s SOT trial to the maximal theoretical sway limits of stability, which was based on the individual’s height and the size of the base of support. It represents the angle (8.0 anteriorly and 4.5 posteriorly) at which the subject could lean in any direction before the center of gravity would move beyond a point that allowed him/her to remain upright (i.e., point of falling). The following formula was used to calculate the ES:

Equilibrium = 12.5° − (Θmax − Θmin)/12.5° × 100

where Θmax indicates the greatest antero-posterior CoG sway angle, and Θmin indicates the lowest antero-posterior CoG sway angle. Lower sways lead to a higher ES, indicating a better balance control performance (a score of 100 represents no sway, while 0 indicates sway that exceeds the limit of stability, resulting in a fall). Table 2 shows the CES and sensory ratios calculation method.

The platform is also designed to register medio-lateral sways, i.e., to appreciate body movements in the coronal plane (“lateral deviation”). When the patient shifts the center of the body medio-laterally from the “zero position”, it is possible to state that the quality of his/her postural control is not optimal due to a deficit of the ipsilateral vestibular system. Even though it is suggested that CDP cannot definitively define the site of lesion [27,34] in gait ataxia, some authors have attempted to distinguish a cerebellar lesion from a vestibular lesion, depending on the posturographic sway pattern [35,36,37], relying primarily on the direction of sway. Maki et al. [35] reported that lateral deviation is predictive of falls in the elderly population. Given this, we decided to also analyze lateral deviation in our population.

CDP also allows us to analyze the strategy adopted by a patient to maintain postural control. The rapid reflex response, which allows the human body to stand up and move with respect to gravity, consists of a muscular reaction in the trunk and legs. In response to an event of equilibrium perturbation, the integration of the three sensorineural afferences produces an organized trunk and leg agonist and antagonist muscles contraction, which maintain the stand-up position [38]. Trunk and leg muscles enable the movement of rigid segments around the ankle, knee, and hip joints. The most ergonomic muscular schema is selected by a healthy human body, and it is preferably driven in an “ascendant direction”, which first implicates the activation of ankle contractions, followed by knee and hip. We consider this strategy as “appropriate”, as oppposed to with “descending strategy”, which involves an initial hip jerk rather than ankle control.

Equitest has been validated as a useful tool to assess balance in children, with a good test–retest reliability [39]. Six years of age is a cut off that has been already identified [40] for the reliability of posturographic examination in the pediatric population.

Group 1 data were compared with the preoperative data from Group 2 to look for any difference between the two populations. Afterward, longitudinal analyses were performed, comparing the preoperative and postoperative data of Group 2 patients.

The parameters of evaluation were as follows: demographic, antenatal and birth data, past medical history, level of schooling, and other everyday life parameters (e.g., ability to ride a bike and confidence on stairs). The subjects also underwent clinical ENT and neurological assessment, MRI, polysomnographic and posturographic tests. They were also asked about any history of headaches or vertigo. In Group 2 children, we compared headaches and vertigo both preoperatively and postoperatively, as this represented the best parameter of postoperative improvement with regard to quality of life. These data were collected using a postoperative written survey.

### 2.3. Statistics

Data analysis was presented as the median with 25th and 75th percentiles (median (interquartile range)) for continuous variables, whereas categorical variables were presented as numbers and percentages. Comparisons of baseline characteristics according to the surgery performed were conducted by using Wilcoxon or Kruskal–Wallis tests for continuous variables and the Fisher exact test or χ^2^ test for categorical variables. Statistical analyses were performed using R, version 4.3.1 (6 June 2016) (R Foundation for Statistical Computing, Vienna, Austria) and Jamovi version 2.4, with a critical level for statistical significance was set at 5%. Significance is expressed on figures and tables as follows: * = *p* < 0.05; ** = *p* < 0.01; *** = *p* < 0.001; NS = *p* > 0.05.

## 3. Results

A total of 28 children (18 boys and 10 girls) aged 6 to 17 years (mean age 11 y) fulfilled the inclusion criteria and were recruited in our study. There were 18 children (5 girls and 13 boys) in Group 1 (non-operated patients) aged between 6 and 17 years. There were 10 children (5 girls and 5 boys) in Group 2 (operated patients) aged between 6 and 16 years. No significant differences were found in age (*p* = 0.904) or sex distribution (*p* = 0.412).

Table 3 reports the epidemiological data of the enrolled patients.

Only 1 patient in Group 2 was born prematurely (i.e., before 37 weeks gestation) and none had a serious prenatal history; and 16 patients (58%) had associated medical pathology unrelated to their CMI, which did not impact their posturographic results.

Twenty-two patients (79%) attended regular school. A total of 15 children (56%) had a history of speech difficulty, necessitating specific therapy; 14 (50%) wore corrective lenses; and 5 (18%) had a previous history of strabismus. Only one child presented with scoliosis; however, no specific radiologic exam has been systematically realized to check for Cobb’s angles. As shown in Table 3, no significant difference between the two groups was found in any of their histories, aside from the ability to ride a bicycle. All of the children in Group 1 were able to ride a bicycle, but 70% of Group 2 patients reported difficulties doing so. This was independent of age (*p* = 0.04).

Three patients (11%) reported a Chiari I malformation history in relatives; however, we did not explore heredity nor genetic aspects.

In seven patients (25%), CMI was discovered serendipitously from a CT scan ordered for minor head trauma. In two patients (7%), it was discovered after a cerebral MRI for developmental delay. In two other patients (7%), it was discovered after MRI ordered for episodic faintness. Twelve children (43%) underwent cerebral imaging for recurrent headaches and two (7%) for nuchal pain. Two children (7%) were investigated for oculomotor trouble and one (4%) for neurological deficit (sensory motor impairment).

Dizziness and scoliosis were not reported as presenting symptoms or signs in any of our cases; however, one patient with headaches reported vertigo as a main presenting symptom, and two patients (7%) had scoliosis found at the initial examination. No patients needed to be treated for scoliosis and none of the aforementioned presenting aspects were found to be significantly different between the two groups (see Table 4).

All but five children complained of headaches, with only one patient describing exertional pain. In 9/28 children (32%), pain was localized to the occipito-cervical region, while all other patients described frontal, fronto-temporal, or holocranial pain. All operated children complained of headaches, but none described exertional ones. The frequency was mostly weekly, without significant difference for any parameter. Nineteen children (68%) had a family history of migraine.

Twelve patients (43%) described having vertigo with different frequencies and characteristics. Interestingly, we found a significant result (*p* = 0.013) comparing the presence of vertigo in the operated versus non-operated patients (respectively, 50% and 6%), with occasional vertigo being more common in the operated patients. These aspects are shown in Table 5.

The evolution of headaches and vertigo in Group 2 patients was evaluated at postoperative follow up, at which time three patients declared a complete resolution of headaches after intervention, with a very low *p*-value (=0.052), even if not significant. It was the number of patients who had “non-nuchal” headaches that better improved, with low *p*-value as well. Two patients had persisting vertigo at the postoperative follow up, compared to six before the intervention; moreover, even if any of the results are statistically significant, it is interesting to notice that positional vertigo disappeared completely after the operation (Table 6).

Generally, all operated patients reported a better quality of life after surgery at the follow-up meetings, due to a reduction or disappearance of such symptoms.

In Table 7, we report data concerning the degrees of cerebral ptosis and polysomnographic results. At cerebral MRI, 12 patients (42%) had a more than 10 mm cerebellar ptosis, 1 patient (3.5%) had ventriculomegaly, and 3 patients (10.5%) had syringomyelia at diagnosis. Seven Group 2 patients (70%) had greater than one-centimeter-sized tonsillar ptosis. This difference is significant. However, one can suggest that the greater the ptosis, the greater the possibility of having a CSF circulation problem at the craniocervical junction. The four children in the series (15% of total population) presenting with central sleep apnea syndrome were operated on, as respiratory problems related to brainstem compression is an absolute criterion for decompressive surgery. For this reason, comparison between the two groups is not useful. Two of them presented a central apnea index superior to 5/h. Fifteen children (56%) presented with obstructive apnea syndrome (OSAS), which is significantly higher than the prevalence in the pediatric age group previously reported by Rydel et al. (10.3% in a population of 399 infants and adolescents from 2 to 18 years of age) [41]. However, further consideration about these two topics is beyond the scope of our paper.

Twelve patients (43%) described having vertigo with different frequencies and characteristics. Interestingly, we found a significant result (*p* = 0.013) comparing the presence of vertigo in operated versus non-operated patients (respectively, 50% and 6%), with occasional vertigo being more common in patients who will be operated on. These aspects are shown in Table 5.

Given the frequent association of audiovestibular symptoms in CMI patients, as discussed above, an ENT assessment was carried out in all children.

The clinical evaluation made by the first author, a neurosurgeon (I.S.), revealed normal neurological status in 13 (72%) Group 1 patients and 3 (30%) Group 2 patients (Table 8). This difference is significant (*p* = 0.049); however, we found that these results may be related to the fact that symptomatology is one of the criteria for surgical intervention. Two children (10%) in Group 2, but none in Group 1, presented with disorders of coordination (ataxia in the upper limbs). Balance disorders were diagnosed by widening of the support polygon, inferior limb ataxia, a positive Romberg’s test, or a history of frequent falls. They were found in two Group 1 patients (11%) and in two Group 2 patients (10%) (not significant). Pyramidal signs were slightly less frequent in Group 1 (18%) than in Group 2 (22%).

From the ENT point of view, 10 patients (56%) in Group 1 and 2 patients in Group 2 (22%) had normal otoneurological examinations. Data are lacking for one operated patient (n° 9), as shown in Table 7, which shows neuro-otological findings. Only one patient in Group 1 presented with clinical vestibular disease. Nystagmus was found in only one patient (11%) in Group 2, while saccadic pursuit was observed in three Group 1 patients (17%) and four Group 2 patients (44%). Although not technically significant, a slightly low *p*-value (0.093) was found comparing the presence of dizziness (33%). Subnormal thresholds in pure-tone conventional audiometry were noted in four patients in Group 1 (22%) and two patients in Group 2 (33%). These results are not statistically different.

Equitest^®^ dynamic posturographic evaluation has been utilized as a complement to ENT investigations. The mean SOT in the whole population was 65.6 (range 65.3–76.5); in addition, when the two groups are separately evaluated, the mean SOT in Group 1 was 66 (64.0–76.0), and in Group 2 it was 62.3 (66.0–77.5), without statistical differences (*p* = 0.655), as shown in Table 9.

Interestingly, we found significant results comparing lateral deviation attitude during posturography, with 17% of abnormal lateral CoG deviation in Group 1, versus 60% in Group 2.

When the same parameters were studied in Group 2, comparing pre and post surgery, a significant difference was seen in the global SOT results (*p* = 0.007). In particular, we found that the sensorineural component of postural control that improved the most was the vestibular one, with a *p* = 0.007 (see Table 10). Low *p*-values were found comparing somesthetic and visual ratios.

**Figure 2:**
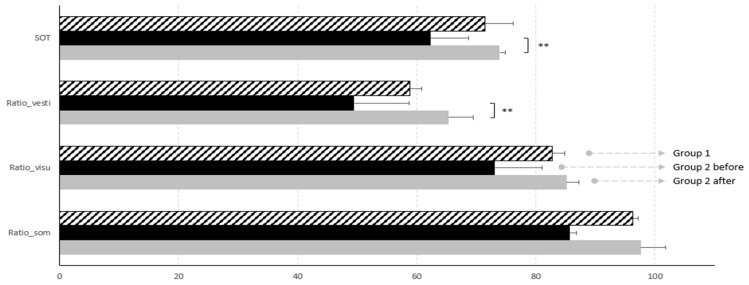
Graphic representation of posturographic results. Global SOT and vestibular ratio improve significantly after surgery. Significance is expressed as follows: ** = *p* < 0.01.

A graphic representation of such results is reported in Figure 2.

**Figure 3:**
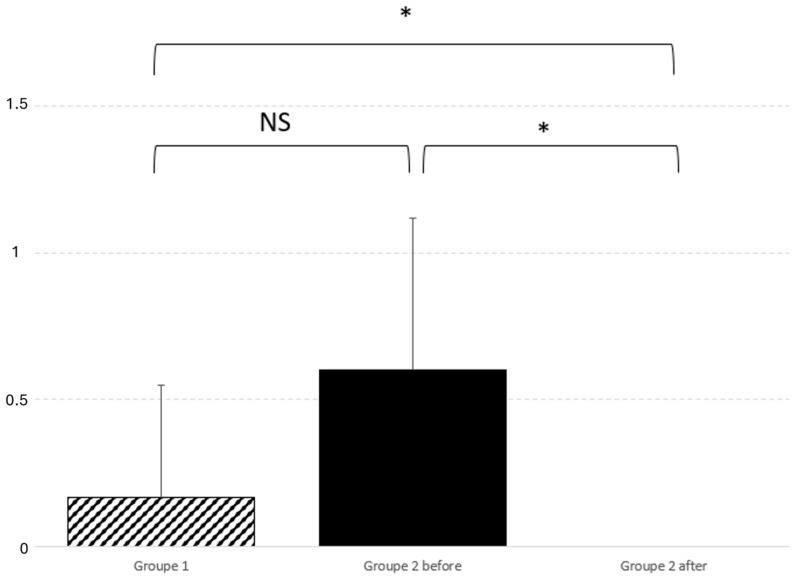
Graphic representation of lateral deviation comparison and significant postoperative difference. Comparison between the two groups did not find any significant difference; *p* = 0.35. On the contrary, real improvement was noticed between before and after the intervention; *p* = 0.011. Significance is expressed as follows: * = *p* < 0.05 and NS = *p* > 0.05.

Also, lateral deviation improves significantly after surgery (*p* = 0.01), as graphically reported in Figure 3.

**Figure 4:**
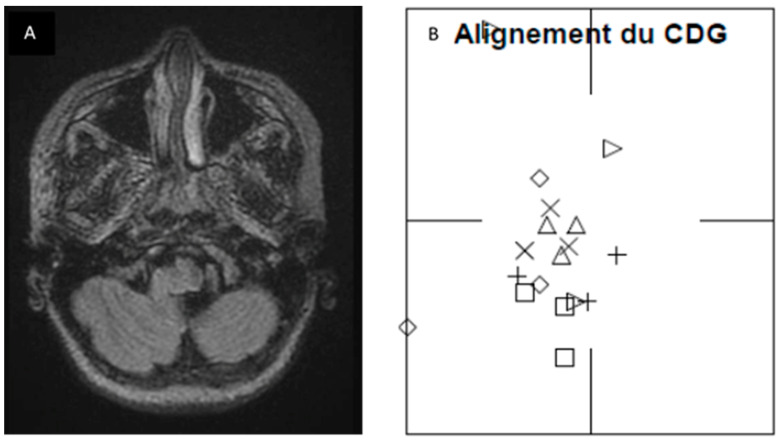
Correspondence between FLAIR MRI tonsillar signal anomaly and lateral deviation on posturography was found in four patients of Group 2. (**A**) FLAIR axial images at craniovertebral junction of patient number 26, showing sufferance signal of left tonsil, going toward the median line. (**B**) Posturographic results showed a deviation of CoG toward the left side (alignment du CDG: center of gravity alignment; CoG: center of gravity). Each shape in subfigure B corresponds to each sensory condition: 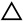: condition 1; 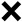: condition 2; 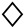: condition 3; 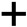: condition 4; 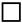: condition 5; 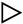: condition 6.

Lateral deviation also improved significantly after surgery (*p* = 0.01), as illustrated in Figure 2. In order to further our knowledge about this finding, we retrospectively checked the preoperative MRI and surgical reports of the operated patients. Although we cannot establish any significant correlation, we found that, in four patients, there was a perfect correspondence between the side of posturography lateralization, MRI 3D FLAIR signal tonsillar anomaly, and the side of tonsillectomy (which normally corresponds to the greater descending tonsil on MRI). Figure 4 shows the pre- and postoperative results of one of the patients. Two other patients, who presented a little MRI FLAIR bilateral tonsillar anomaly, had a normal CoG without any lateralization, and one of them benefited from minimal bilateral tonsillar coagulation.

However, the small number of patients included in our series did not allow us to perform a statistical analysis for this aspect. We are increasing the number of patients in our study to try and confirm our speculations.

## 4. Discussion

The results showed a significant improvement of global SOT after intervention (*p* = 0.007), leading us to believe that surgical treatment of Chiari malformation improves postural control in the pediatric population. When the three sensorial afferences determining postural control were independently considered, comparison between pre and post intervention showed low *p*-values for somesthetic and visual ratios, but a significant difference (*p* = 0.005) for the vestibular one. These data suggest that the most impacted system in CMI children is the vestibular system.

Interestingly, we also observed lateral deviation on the preoperative CDP in the Group 2 patients, which significantly differed from that of Group 1 patients. This lateral deviation reduced significantly after intervention in the Group 2 patients, increasing the value of our findings. This finding may be of particular importance, as it suggests that there is a correspondence between the direction of lateral deviation and the side of tonsillar signal anomaly on 3D FLAIR sequences. If such a correspondence can be confirmed, axial 3D FLAIR images and CDP could be included in the standardized workup of CMI patients, as it would be of help in making a surgical decision. Further work is needed to corroborate this finding, as our numbers are too low to show significance.

From a neurosurgical point of view, it is crucial for us to know what might be related to CMI in order to assure the best care for our patients. In CMI patients who manifest signs or symptoms clearly related to the malformation (i.e., central sleep apnea syndrome, syringomyelia, effort headaches, and tetra paresis), the need for surgical treatment is rapidly established. However, vestibular assessment may become an essential assessment in a category of CMI patients called “asymptomatic” or “oligo symptomatic”, because of the absence of typical CMI symptoms. Many papers have been published on this subject, but the cause and effect relationship between the otoneurological signs and symptoms and CMI still remains a source of debate in the neurosurgical community [42]; in addition, this can create controversy regarding surgical management, as these symptoms and signs are not historically directly attributed to the presence of CMI [43] and do not provide sufficient information for the neurosurgeon to make a decision about surgical intervention. However, in a recent special journal issue, these ENT symptoms have been reported as possible CMI manifestations and have been defined as ‘atypical’ symptoms by Novegno et al. [44]. For this reason, an ENT assessment should be required for all CMI patients.

In the literature, we found only two papers [29,30] that studied the relationship between CMI and posturographic assessment; however, neither study reported any information about the lateral sway pattern, which suggests that our study is the first one to look at lateral deviation in children with CMI.

Famili [29] studied Equitest^®^ results in a cohort of 24 CMI adults (21/24 females, aged from 20 to 61 years) who complained about dizziness symptoms. The data suggested a significantly lower use of somatosensory information, but a peripheral vestibular lesion was found in only 8% of patients, suggesting a central origin of dizziness in this population.

**Figure 5:**
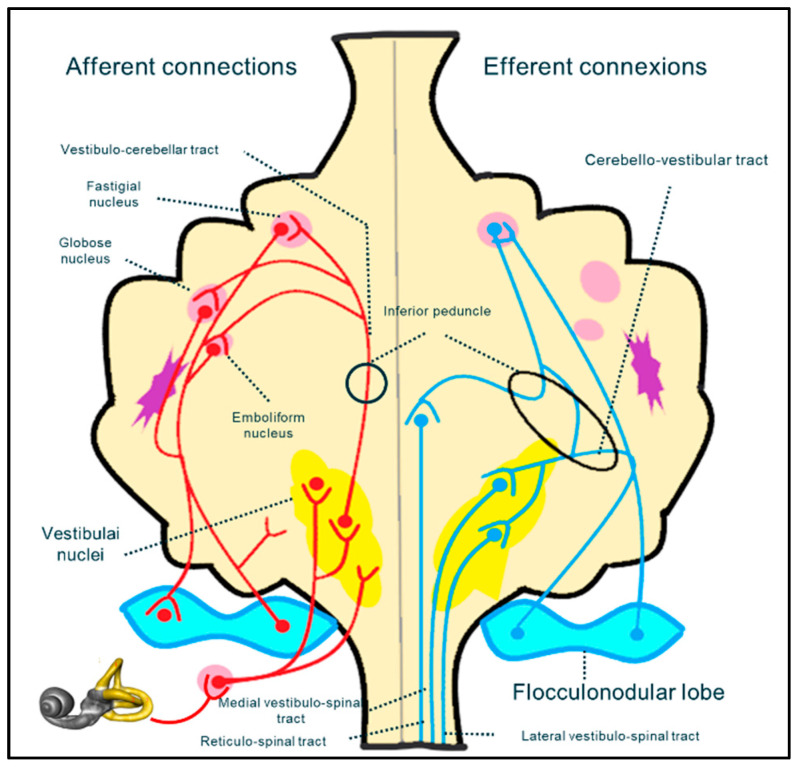
Schematic reconstruction of the neural pathway implicated in the sensory neural integration for postural control.

It is difficult to determine (especially in the pediatric population) whether the vestibular impairment in CMI results from damage to afferent fibers of the spinocerebellar bundle, from cranial nerve dysfunction (in the central or peripheral portion), or from the cerebellar vestibular nuclei. The vestibular system is a complex neuronal pathway whose central component is situated mostly in the posterior fossa, at the level of the craniocervical junction (Figure 5). It includes the cerebellar nuclei and cranial nerve nuclei in the brainstem. The peripheral portion of the vestibular system includes the VIIth cranial nerve, which leaves the brainstem to reach its destination through the acoustic pore.

Direct compression on the flocculo-nodular lobe, spinocerebellar and cerebello-vestibular tracts, or vestibular nuclei, or distortion of vestibular nerves could produce balance problems and ataxia. In addition, at the same level, olivary nuclei are interlaced in the pretectal flocculo-nodular pathway, which is implicated in the modulation of the visual information that is essential for balance control (e.g., optokinetic nystagmus and ocular pursuit) [45].

A hypothesis has been proposed [9] that a central origin of vestibular impairment in CMI patients is most likely. This is consistent with other reports [29,46]. Moncho et al. [42,47] showed an abnormal brainstem auditory-evoked potential (BAEP) in a cohort of 200 CMI adult patients, mainly at the retrocochlear level, suggesting the implication of CMI-related brainstem distortion (i.e., a central origin). A recent study of patients with endolymphatic hydrops (i.e., a peripheral vestibulopathy) showed problems with perilymph–CSF dynamics due to CSF hydrodynamic alterations at the foramen magnum level and suggested that this may be a possible mechanism of ENT-related symptoms in CMI patients [20].

In our study, a complete neuro-otological examination, accompanied by audiometry and further management, if deemed necessary, was carried out on all of the children. However, given the limited cooperation of children, we did not include systematic complex instrumental ENT examination, even though that would have helped us to delineate brainstem involvement from peripheral vestibular involvement [17]. A lack of cooperation, mental disability, behavioral trouble, orthopedic disease, and visual impairment are all factors that might influence the CPD results. For this reason, children presenting with any of these issues were not included in this study.

Children generally have fewer comorbidities than adults, and they do not normally present with degenerative body issues that might also affect postural control. In our study, none of the operated children showed clinical vestibular impairment before intervention, thus reinforcing the idea that postoperative vestibular improvement detected on CPD is directly linked to craniovertebral junction decompression rather than from other confounding factors.

A lack of cooperation is one of the most important limiting factors in a clinical study in children of this age, as it narrows the wide spectrum of available tests for the study of the vestibular system, which reduces the precision of the assessment in such a population. In addition, we feel it is important in this discussion to outline the specificity of the development of the vestibular system in the pediatric population. This will also help us to come to some conclusions regarding our results.

Sensory inputs utilized by the balance system (somesthetic, visual, and proprioceptive inputs) change progressively during development [40,48,49,50,51]. Sinno et al. [51] recently studied 120 healthy children utilizing Equitest^®^, reported SOT normative data throughout childhood and adolescence, and outlined how postural abilities change during development. Compared to adults, younger children utilize more somatosensory inputs. Even though the vestibular system is the first system to develop and is morphologically complete at birth, vestibular maturity is reached later, as it entails a higher processing center of neurosensory inputs utilized for postural control. In addition, balance performance declines not only between the young and the elderly, but also from decade to decade, as demonstrated by the Polish team of Pierchala et al. [52].

The age difference may be one of the reasons for the fact that our results are in contrast with the posturographic results in adults reported by Famili [29]. In addition, that study was characterized by a high prevalence of females (90%), and one might suggest that this gender distribution imbalance might have influenced their results. In our study, gender distribution was equal in Group 2, although there was a slight female prevalence (66%) in the overall population. There are few papers directly addressing gender influence on CDP results [53], but some studies [48,49,50,54] have also reported this information, while focusing primarily on aged-related modifications. It is generally assumed that, with increasing age, scores worsen in both males and females in more difficult CDP conditions (Conditions 5 and 6) [55,56]. In a study conducted by Faraldo-Garcia et al. [53], females seemed to be better at integrating sensory vestibular information than males, as well as at dealing with visual conflict.

One may also ask if learning phenomena or age-related bias might influence our results. Given the structure of our study [28], age-related improvement is not a factor, as posturographies were performed 2 to 3 months before and 3 to 4 months after surgical intervention. This laps of time is not enough to observe dramatic improvement of complex sensory integration ability due to the child’s growth. In the same way, it is too long to significantly impact memory, so it cannot explain SOT postoperative improvement.

In our study, the SOT and vestibular ratio improvement observed after surgery for CM1 leads us to believe that posterior fossa overcrowding in this disorder disturbs the postural abilities, predominantly affecting the vestibular system. As the most significant limit of our study is the small sample size, we are continuing our study so that our sample size can be increased.

## 5. Conclusions

The surgical indication for CMI patients may be obvious when concerning symptoms and signs, such as syringomyelia or sleep apnea syndrome, are present. However, decisions made regarding surgical management may be more controversial when patients are asymptomatic or when they present with “atypical” symptoms and signs, such as vestibular and postural complaints. Due to the increasing awareness about the existence of these atypical symptoms in CMI, a specific ENT evaluation is highly recommended as part of a preoperative workup. This is particularly important in children, as it is even more difficult to determine the presence of symptoms linked to CMI, and also more difficult to take an accurate history to clarify their signs and symptoms.

Computerized dynamic posturography may be a good solution to examine ataxia and postural patterns in children with CMI over 6 years of age, as it is a safe and sensitive measuring tool. However, further studies with a greater number of patients are necessary to better elucidate the role of CDP in the workup of CMI patients.

## Figures and Tables

**Table 1:** Computerized dynamic posturography. Sensory organization test (Equitest^®^, NeuroCom, Clackamas, OR, USA). Determination of the six conditions. SR, sway-referenced [27,31,32,33].

Postural Control Test		
Name	Situation	Sensory Consequences
Condition 1	Fixed support, eyes open	-
Condition 2	Fixed support, eyes closed	Vision absent
Condition 3	Fixed support, SR surround	Altered vision
Condition 4	SR support, eyes open	Altered proprioception
Condition 5	SR support, eyes closed	Vision absent, altered proprioception
Condition 6	SR support, SR surround	Altered vision and proprioception

**Table 2:** Computerized dynamic posturography. Sensory organization test (Equitest^®^, NeuroCom, Clackamas, OR, USA). Significance of composite score and sensory ratios.

Name	Equation	Significance
Composite score	[C1 + C2 + 3 (C3 + C4 + C5 + C6)]/14	Evaluate global balance performance. A low score represents poor postural control.
Somatosensory ratio	C2/C1	Ability to use somatosensory input to maintain balance (even when visual cues are removed). A low score suggests poor use of somatosensory references.
Visual ratio	C4/C1	Ability to use visual input to maintain balance (even when somatosensory cues are altered). A low score suggests poor use of visual references.
Vestibular ratio	C5/C1	Ability to use vestibular input system to maintain balance (even when visual cues are removed and somatosensory cues are altered). A low score suggests poor use of vestibular cues or that vestibular information is unavailable.
Visual preference ratio	C3 + C6/C2 + C5	Degree to which patient relies on visual information to maintain balance (correct/incorrect information). A low score suggests reliance on visual cues even when they are inaccurate.

**Table 3:** Epidemiological and basic medical information of the 18 included patients. In some cases, data are lacking for one patient. Fisher’s exact test was utilized for statistical analysis for all variables except the wearing of glasses (Chi square test). Significance is expressed as follows: * = *p* < 0.05.

Variable	Total Population (*n* = 28/27)	Group 1 (*n* = 18/17)	Group 2 (Before Operation) (*n* = 10)	*p*-Value
Genre male	18 (64%)	13 (72%)	5 (50%)	0.26
Age	10.0 (8.0–14.0)	10.0 (8.0–13.0)	10.0 (8.0–15.0)	0.90
Normal scholarship	22/28 (79%)	14/18 (78%)	8/10 (80%)	1.00
Chronic non-neurologic illness	6/28 (21%)	5/18 (28%)	1/10 (10%)	0.37
Non-chronic, non-neurologic anterior medical history	4/27 (15%)	1/17 (6%)	3/10 (30%)	0.13
Non-chronic, non-neurologic anterior surgery	6/27 (22%)	3/17 (18%)	3/10 (30%)	0.64
Genetic pathology	2/27 (7%)	1/17 (6%)	1/10 (10%)	1.00
Neurologic pathology	1/27 (4%)	1/17 (6%)	0/10 (0%)	1.00
ENT non-vestibular pathology	1/28 (4%)	1/17 (6%)	0/10 (0%)	1.00
Orthopedic pathology	1/28 (4%)	1/17 (6%)	0/10 (0%)	1.00
Respiratory pathology	2/28 (7%)	2/17 (11%)	0/10 (0%)	0.52
Strabismus	5/28 (18%)	3/17 (17%)	2/10 (20%)	1.00
Speech difficulties/Speech therapy	15/27 (56%)	8/17 (47%)	7/10 (70%)	0.42
Glasses	14/27 (50%)	8/17 (44%)	6/10 (60%)	0.43
**Bicycle driving difficulties**	**24/28 (89%)**	**18/18 (100%)**	**7/10 (70%)**	0.041 *

**Table 4:** Presenting symptoms of enrolled patients, leading to cerebral imaging. The first presenting symptom was headache, but the second most important way to diagnose was accidental finding (Fisher’s exact test).

Presenting Symptom	Total Population (*n* = 28)	Group 1 (*n* = 18)	Group 2 (Before Operation) (*n* = 10)	*p*-Value
Headaches	12 (43%)	6 (33%)	6 (60%)	0.24
Accidental	7 (25%)	4 (22%)	3 (30%)	0.67
Developmental delay	2 (7%)	2 (11%)	0 (0%)	0.52
Malaise	2 (7%)	2 (11%)	0 (0%)	0.52
Nuchal pain	2 (7%)	1 (6%)	1 (10%)	1.00
Oculomotor deficit	2 (7%)	2 (11%)	0 (0%)	0.52
Sensory-motor deficit	1 (4%)	1 (6%)	0 (0%)	1.00

**Table 5:** Headaches and vertigo characteristics in the enrolled patients. Results based on patients’ survey. Globally, headaches are declared by 82% of the enrolled patients, and in all operated children. No patients but one Group 1 patient declared exertional headaches. No significant differences were found between the two groups. Concerning vertigo, prevalence in the total population was 43%, being 60% in Group 2. Significant difference is noted in vertigo frequency (Fisher’s exact test). Significance is expressed as follows: * = *p* < 0.05.

Headaches and Vertigo		Total Population	Not Operated	Operated	*p*-Value
Headaches	Global	23/28 (82%)	13/18 (72%)	10/10 (100%)	0.128
	Occipital headaches	9/28 (32%)	5/18 (28%)	4/10 (40%)	0.68
	Non-occipital headaches	14/28 (50%)	9/18 (6%)	6/10 (60%)	1.00
	Exertional headaches	1/28 (4%)	1/18 (6%)	0/10 (0%)	1.00
	Weekly headaches	17/28 (61%)	10/18 (56%)	7/10 (70%)	0.69
	Monthly headaches	6/28 (21%)	3/18 (17%)	3/10 (30%)	0.63
	Drugs resolving headaches	12/27 (44%)	6/17 (35%)	6/10 (60%)	0.26
	Rest and dark resolving headaches	4/27 (15%)	2/17 (12%)	2/10 (20%)	0.61
	Family migraine	19/28 (68%)	12/18 (67%)	7/10 (70%)	1.00
	Associated vomiting	6/27 (22%)	3/17 (18%)	3/10 (30%)	0.64
Vertigo	Global	12/28 (43%)	6/18 (33%)	6/10 (60%)	0.24
	Weekly vertigo	6/28 (21%)	5/18 (28%)	1/10 (10%)	0.37
	**Monthly vertigo**	**6/28 (21%)**	**1/18 (6%)**	**5/10 (50%)**	0.013 *
	Positional vertigo	7/28 (25%)	4/18 (22%)	3/10 (30%)	0.67
	Exertional vertigo	4/28 (14%)	1/18 (6%)	3/10 (30%)	0.12
	Vision-related vertigo	1/28 (4%)	1/18 (6%)	0/10 (0%)	1.00
	Motion sickness	7/28 (25%)	5/18 (28%)	2/10 (20%)	1.00

**Table 6:** Evolution of headaches and vertigo after the intervention in Group 2 patients. Even if any of the results are significant from a statistical point of view, we found a really low *p*-value comparing preoperative and postoperative headaches (*p* = 0.052), especially for non-nuchal pain (*p* = 0.059).

Group 2	Before Intervention	After Intervention	*p*-Value
Headaches	10/10 (100%)	7/10 (70%)	*p* = 0.052
Nuchal	4/10 (40%)	1/7 (14%)	*p* = 0.527
Non-nuchal	6/10 (60%)	6/7 (71%)	*p* = 0.059
Vertigo	6/10 (60%)	2/10 (20%)	*p* = 0.593
Positional	3/10 (30%)	0/2	*p* = 0.655
Exertional	3/10 (30%)	1/2 (50%)	*p* = 0.317
Brutal	0/10	1/2 (50%)	*p* = 0.317

**Table 7:** (A) Radiologic details concerning the degree of cerebellar ptosis and presence of syringomyelia. (B) Polysomnography results. Significant difference is obviously found, because syringomyelia and CSAS are absolute criteria to operate (Fisher’s exact test). In the same manner, the most important factor is the degree of tonsillar ptosis. A higher degree is related to an increased probability of the patient developing CSF circulation pathology at the craniocervical junction. In Group 1, polysomnographic data are missing for one patient. AHI: Apnea-hypopnea index; OAHI: Obstructive Apnea-hypopnea index. Significance is expressed as follows: * = *p* < 0.05 and ** = *p* < 0.01.

	Variable	Total Population (*n* = 28/27)	Group 1 (*n* = 18/17)	Group 2 (Before Operation) (*n* = 10)	*p*-Value
**A**	**Tonsillar ptosis > 1 cm**	12/28 (43%)	5/18 (28%)	7/10 (70%)	0.049 *
	**Syringomyelia**	4/28 (14%)	0 (0%)	4/10 (40%)	0.010 **
**B**	**Central apnea syndrome**	4/27 (15%)	0/17 (0%)	4/10 (40%)	0.012 *
	**AHI > 5/h**	2/27 (7%)	0/17 (0%)	2/10 (20%)	0.13
	**Obstructive apnea syndrome**	15/27 (56%)	9/17 (53%)	6/10 (60%)	1.00
	**OAHI > 5**	2/27 (7%)	2/17 (12%)	0/10 (0%)	0.52

**Table 8:** Neurologic and audiovestibular findings in the enrolled patients. In the group of operated patients, data are lacking for one patient. Neurological examination was carried out by the first author (I.S.), while ENT assessment was realized by the second author (Ph.P.). A significant difference was found when comparing the two groups with regard to the presence of sensory disorders, and a low *p*-value was shown when comparing the presence of dizziness between the two groups (Fisher’s exact test). Significance is expressed as follows: * = *p* < 0.05 and ** = *p* < 0.01.

	Variable	Total Population (*n* = 28/27)	Group 1 (*n* = 18/17)	Group 2 (Before Operation) (*n* = 10)	*p*-Value
**Neurological**	**Normal**	**16/28 (57%)**	**13/18 (72%)**	**3/10 (30%)**	0.049 *
	Coordination disorders	2/28 (7%)	0/18 (0%)	2/10 (20%)	0.12
	Balance disorders (ataxia)	4/28 (14%)	2/18 (11%)	2/10 (20%)	0.60
	OTR anomalies	6/28 (21%)	4/18 (22%)	2/10 (20%)	1.00
	**Sensitive disorders**	**4/28 (14%)**	**0/18 (0%)**	**4/10 (40%)**	0.010 **
	Oculomotor trouble	1/28 (4%)	0/18 (0%)	1/10 (10%)	0.36
ENT	Normal	12/27 (44%)	10/18 (56%)	2/9 (22%)	0.22
	Vestibular deficit	1/27 (4%)	1/18 (6%)	0/9 (0%)	1.00
	Nystagmus	1/27 (4%)	0/18 (0%)	1/9 (11%)	0.33
	Saccadic eye movements	7/27 (26%)	3/18 (17%)	4/9 (44%)	0.18
	Dizziness	4/27 (15%)	1/18 (6%)	3/9 (33%)	0.093
	Hearing loss	7/27 (26%)	4/18 (22%)	3/9 (33%)	0.65
	Tinnitus	3/27 (11%)	1/18 (6%)	2/9 (22%)	0.25

**Table 9:** Posturographic results of Group 1 patients and Group 2 patients before the intervention. No significant difference is found comparing global SOT and the 3 ratios. On the contrary, lateral CoG deviation is significantly different between the 2 groups (Wilcoxon–Mann–Whitney test; §: Fisher’s exact test).

Variable	Total Population (*n* = 28)	Group 1 (*n* = 18)	Group 2 (Before Operation) (*n* = 10)	*p*-Value
**SOT**	65.6 (65.3–76.5)	66 (64–76)	62.3 (66–77.5)	0.655 §
**Somesthetic Ratio**	97.0 (91.5–98.5)	97.5 (95.0–98.0)	95.0 (68.1–99.0)	0.37 §
**Visual Ratio**	85.5 (74.5–91.5)	86.0 (79.0–91.0)	85.0 (65.3–95.0)	0.98
**Vestibular Ratio**	60.0 (40.5–69.0)	61.0 (41.0–69.0)	56.4 (39.0–64.0)	0.41
**Lateral deviation**	9 (32%)	3 (17%)	6 (60%)	0.35
**Inappropriate strategy**	11 (39%)	6 (33%)	5 (50%)	0.44

**Table 10:** Preoperative and postoperative posturographic results in the Group 2 patients. Significant differences were found comparing global SOT and vestibular ratio. Interestingly, low *p*-values were found when comparing somesthetic and visual ones. In the same way, lateral CoG deviation is also significantly different (Wilcoxon–Mann–Whitney test; §: Fisher’s exact test). Significance is expressed as follows: * = *p* < 0.05 and ** = *p* < 0.01.

Group 2 (*n* = 10)	Before Surgery	After Surgery	*p*-Value
**SOT**	**62.3 (66–77.5)**	**73.9 (67–80.8)**	0.007 § **
**Somesthetic ratio**	85.7 (73.5–98.5)	97.6 (95.3–99.5)	0.096 §
**Visual ratio**	73.1 (68.5–94.3)	85.1 (74.8–93.5)	0.084 §
**Vestibular ratio**	**49.5 (42–63)**	**65.3 (60.5–77.5)**	0.005 § **
**Lateral deviation**	**6/10 (60%)**	**0/10 (0%)**	0.011 *
**Inappropriate strategy**	5/10 (50%)	1/10 (10%)	0.141

## Data Availability

Data are available on the Nancy Regional Hospital Center database and are only known by the principal investigator due to the CNIL privacy policy.
